# The Effects of Coarse Particles on Daily Mortality: A Case-Crossover Study in a Subtropical City, Taipei, Taiwan

**DOI:** 10.3390/ijerph13030347

**Published:** 2016-03-22

**Authors:** Meng-Hsuan Cheng, Hui-Fen Chiu, Chun-Yuh Yang

**Affiliations:** 1 Division of Pulmonary and Critical Medicine, Department of Internal Medicine, Kaohsiung Medical University Hospital, Kaohsiung 807, Taiwan; cmhkmu@gmail.com; 2 Graduate Institute of Medicine, Kaohsiung Medical University, Kaohsiung 807, Taiwan; 3 Department of Pharmacology, College of Medicine, Kaohsiung Medical University, Kaohsiung 807, Taiwan; chiu358@yahoo.com.tw; 4 Department of Public Health, College of Health Sciences, Kaohsiung Medical University, Kaohsiung 807, Taiwan; 5 Division of Environmental Health and Occupational Medicine, National Health Research Institute, Miaoli 350, Taiwan

**Keywords:** coarse particulate, air pollution, daily mortality, case-crossover

## Abstract

Many studies have examined the effects of air pollution on daily mortality over the past two decades. However, information on the relationship between levels of coarse particles (PM_2.5–10_) and daily mortality is relatively sparse due to the limited availability of monitoring data. Furthermore, the results are inconsistent. In the current study, the association between coarse particle levels and daily mortality in Taipei, Taiwan’s largest city, which has a subtropical climate, was undertaken for the period 2006–2008 using a time-stratified case-crossover analysis. For the single pollutant model (without adjustment for other pollutants), PM_2.5–10_ showed statistically significant association with total mortality both on warm and cool days, with an interquartile range increase associated with a 11% (95% CI = 6%–17%) and 4% (95% CI = 1%–7%) rise in number of total deaths, respectively. In two-pollutant models, PM_2.5–10_ remained significant effects on total mortality after the inclusion of SO_2_ and O_3_ both on warm and cool days. We observed no significant associations between PM_2.5–10_ and daily mortality from respiratory diseases both on warm and cool days. For daily mortality from circulatory diseases, the effect of PM_2.5–10_ remained significant when SO_2_ or O_3_ was added in the regression model both on warm and cool days. Future studies of this type in cities with varying climates and cultures are needed.

## 1. Introduction

Over the past decade, many epidemiologic studies demonstrated positive associations between ambient levels of airborne particulate matter (PM) (generally measured as PM with an aerodynamic diameter ≤10 μm [PM_10_]) and daily mortality rate [1,2,3,4,5,6,7] and hospital admissions or emergency room (ER) visits for cardiovascular and respiratory morbidity [8,9,10,11,12]. The evidence for adverse effects of PM air pollution on public health has led to more stringent standards for levels of PM in outdoor air in the USA and other countries [13].

While previous studies have primarily used PM_10_ as an exposure indicator, fine particles (PM_2.5_) have become a greater health and regulatory concern due to epidemiologic studies suggesting that PM_2.5_ might exert more severe damage than larger particles [14,15,16,17]. It is now generally accepted that PM_2.5_ are more harmful to health than larger particles (PM_10_) because PM_2.5_ offer a greater surface area and hence potentially larger concentrations of adsorbed or condensed toxic air pollutants per unit mass [18,19]. Indeed, this provides the basis for the World Health Organization (WHO) recommendation to use PM_2.5_ rather than PM_10_ concentrations as air quality indicators [7].

Most prior studies have only used PM_2.5_ or PM_10_ as PM measurement, meaning that the effects of other particle sizes—particularly PM_2.5–10_—are not well understood [19,20,21]. Fewer studies have examined the potential adverse health effects attributed to the coarse fraction, that is, particles ranging in size from 2.5 μm to 10 μm in aerodiameter (PM_2.5–10_) [20]. PM_2.5–10_ originate mainly from abrasive mechanical processes such as mechanical grinding, windblown dust, and agricultural activities. PM_2.5–10_ are predominantly composed of crustal-related materials such as calcium (Ca), magnesium (Mg), aluminum (Al), silicon, and iron (Fe), and primary organic materials such as pollen, spores, as well as plant and animal debris [18,22,23]. In contrast, the origin of chemical composition of PM_2.5_ is combustion-related constituents. PM_2.5_ are composed of many organic and inorganic compounds, including sulfate, nitrate, organic carbon and elemental carbon, carbonates, metals, and water [24,25]. The adverse health effects associated with ambient exposure to PM_2.5–10_ may thus differ from those of PM_2.5_ considering differences in the sites of deposition in the respiratory tract and sources and chemical composition for these two different-sized fractions [26].

Relatively fewer studies have been undertaken which address the association between PM_2.5–10_ and rate of mortality because only a few cities have monitoring data [16,21,27,28,29,30,31]. Not surprisingly, findings with PM_2.5–10_ are thus inconsistent. These studies were conducted primarily in America and European cities [16,27,29,30,31], with only two studies conducted in Asia [21,28]. As a result, the findings may not be applicable to Asian countries such as Taiwan, where the characteristics of study context may be different, such as levels of PM_2.5–10_, population sensitivity to PM_2.5–10_, variability in PM_2.5–10_ composition and toxicity [32]. Thus, more comprehensive knowledge of the health risks associated with exposure to PM_2.5–10_ is still needed, as suggested in a review on this topic by Brunekreef and Forsberg [20].

To our knowledge, no Taiwanese epidemiological studies investigated the acute effects of PM_2.5–10_ due to the lack of monitoring data. This study was thus undertaken to examine the association between short-term exposure to PM_2.5–10_ and daily mortality among individuals residing in Taipei city, the largest metropolitan city in Taiwan, over a 3-year period from 2006 to 2008, using a case-crossover design.

## 2. Materials and Methods 

### 2.1. Taipei City

This study examined daily mortality in relation to PM_2.5–10_ levels in Taipei for the 3-year period from 2006 to 2008. Taipei is the largest metropolitan city in Taiwan with a population of approximately 2.64 million located in northern Taiwan. The major air pollution source is automobile exhaust emission. Taipei has a subtropical climate, with an annual average temperature of 23 °C.

### 2.2. Mortality Data

Total daily deaths within Taipei city were obtained from the Department of Health (DOH), which is in charge of the death registration system, for the period from 2006 to 2008 (daily mortality data from DOH were not available nationwide after the year 2009). For each death, detailed demographic information, including gender, date of death, date of birth, cause of death, place of death, and residential district were recorded. Deaths due to accidents (ICD-9 codes 800–999) and deaths occurring outside of the city were excluded from the analysis. The deaths were divided into the following two groups according to the International Classification of Diseases, 9th revision (ICD-9): (1) diseases of the respiratory system (ICD-9 codes 460–519); and (2) diseases of circulatory systems (ICD-9 codes 390–459). Further, this study was approved by the ethics review board of Kaohsiung Medical University Hospital (KMUHIRB-EXEMPT-20150016).

### 2.3. Pollutants and Meteorological Data

**Figure 1:**
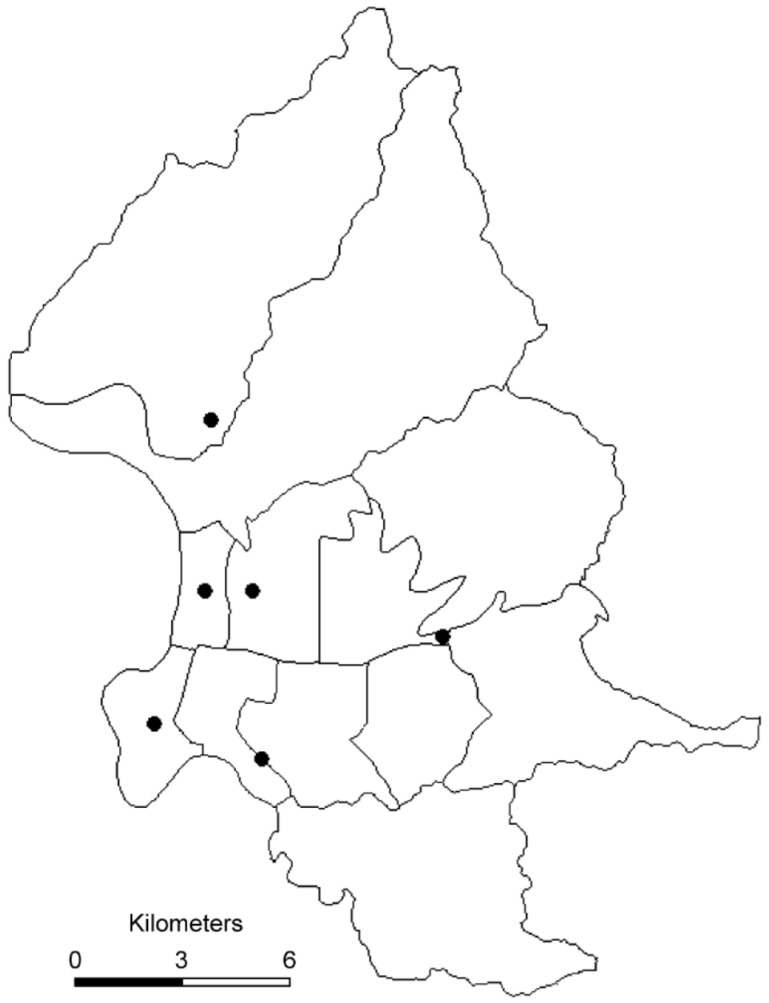
Map of Taipei city showing location of the air-quality-monitoring stations.

Six air quality monitoring stations were established in Taipei city by the Taiwanese Environmental Protection Administration (EPA), a central governmental agency in 1994 (Figure 1). The monitoring stations were fully automated and routinely monitored five “criteria” pollutants including sulfur dioxide (SO_2_) (by ultraviolet fluorescence); particulate matter (PM_10_) (by beta-ray absorption); nitrogen dioxide (NO_2_) (by ultraviolet fluorescence), carbon monoxide (CO) (by nondispersive infrared photometry), and ozone (O_3_) (by ultraviolet photometry) levels. However, PM_2.5_ was not regularly monitored. PM_2.5_ concentrations in Taiwan were measured continuously since 2006. PM_2.5_ was measured using tapered element oscillating microbalance method samplers. The availability of the monitoring network for PM_2.5_ and the continuation of PM_10_ monitoring provided an opportunity to calculate PM_2.5–10_ concentrations. The concentration of the coarse fraction was calculated by subtracting PM_2.5_ levels from PM_10_ levels. For each day, hourly air pollution data were obtained for all of the monitoring stations. We calculated the daily 24-h mean concentrations for PM_10_, PM_2.5_, PM_2.5–10_, NO_2_, CO, and SO_2_ and maximal 8-h mean concentration for O_3_. The maximal 8-h mean was used because the WHO recommended that the 8-h mean reflects the most health-relevant exposure to O_3_ [7]. Daily information on mean temperature and mean humidity was provided by the Taipei Observatory of the Central Weather Bureau.

### 2.4. Statistics

Data were analyzed using the case-crossover technique [33,34,35]. This design is an alternative to Poisson time series regression models for studying the short-term effects attributed to air pollutants [36]. In general, the case-crossover design and the time-series approach yielded almost identical results [37,38,39].

The time-stratified approach was used for the case-crossover analysis [36]. A stratification of time into separate months was made to select referent days as the days falling on the same day of the week within the same month as the index day. Air pollution levels during the case period were compared with exposures occurring on all referent days. This time-stratified referent selection scheme minimizes bias due to non-stationarity of air pollution time-series data [40,41,42]. The results of previous studies indicated that increased mortality was associated with higher air pollutant levels on the same day or the previous two days [43]. Longer lag times have rarely been described. Thus, the cumulative lag period up to 2 previous days (*i.e.,* the average air pollutant levels of the same and previous 2 days) was used. Because pollutants vary considerably by season, especially O_3_ and particles, seasonal interactions between PM and daily mortality have often been reported. However, previous studies were conducted mostly in countries where the climates are substantially different from that in Taipei [44] which has a subtropical climate with no apparent four-season cycle. Hence in this study the potential interactions of seasonality on the effects of PM_2.5–10_ was not considered; but temperature was used instead. The adverse health effects of each air pollutant were examined for the “warm” days (days with a mean temperature above 23 °C) and “cool” days (days with a mean temperature below 23 °C) separately.

The associations between mortality and levels of PM_2.5–10_ were estimated using the odds ratio (OR) and their 95% confidence intervals (CI) which were produced using conditional logistic regression with weights equal to the number of deaths on that day. All statistical analyses were performed using the SAS package (version 9.2; SAS Institute Inc., Cary, NC, USA). Both single-pollutant models and multi-pollutant models were fitted with a different combination of pollutants (up to two pollutants per model) to assess the stability of the effect of PM_2.5–10_. Exposure levels to air pollutants were entered into the models as continuous variables. Meteorologic variables such as daily average temperature and humidity on the same day, which might play a confounding role, were included in the model. Inclusion of barometric pressure did not markedly change the effect estimates and therefore was not considered in the final model. OR were calculated for the interquartile difference (IQR, between the 25th and the 75th percentile) for PM_2.5–10_, as observed during the study period.

## 3. Results and Discussion

The distribution of air pollution, meteorologic measurements, and daily number of deaths in Taipei during the period from 2006 to 2008 are shown in Table 1. An average of 14 persons died of non-accidental causes each day in the city over the study period. Median PM_2.5–10_ during study period was 19.61 ug/m^3^ (IQR: 14.65–24.78).

Pearson’s correlation coefficients among the air pollutants are presented in Table 2. Table 3 shows the effect estimates of PM_2.5–10_ on daily mortality in single-pollutant models and two-pollutant models. For the single pollutant model (without adjustment for other pollutants), PM_2.5–10_ showed statistically significant association with total mortality both on warm and cool days, with an IQR increase associated with a 11% (95% CI = 6%–17%) and 4% (95% CI = 1%–7%) rise in number of total deaths, respectively. There is no indication of an association between PM_2.5–10_ and total number of death due to respiratory diseases both on warm and cool days. PM_2.5–10_ had significant effects on the risk of death from circulatory diseases only on warm days (10% increase for each IQR range change; 95% CI = 1%–21%).

In two-pollutant models, PM_2.5–10_ remained significant effects on total mortality after the inclusion of SO_2_ and O_3_ both on warm and cool days. We observed no significant associations between PM_2.5–10_ and daily mortality from respiratory diseases both on warm and cool days. For daily mortality from circulatory diseases, the effect of PM_2.5–10_ remained significant when SO_2_ or O_3_ was added in the regression model both on warm and cool days.

This study is one of the few that investigated the association between exposure to PM_2.5–10_ and daily mortality in Asia [21,28]. Consistent with previous studies, our study demonstrated significant association between PM_2.5–10_ and the risk of mortality for all causes both on warm and cool days in Taipei [16,27,31].

The correlation between fine and coarse PM was moderate at values of 0.28–0.69 in previous studies with higher value at 0.69 in Steubenville, USA [45]. In contrast, the relationship between PM_10_ and fine as well as coarse PM was greater [20]. The implication is that analyses based on PM_10_ are generally not able to support arguments on the relative importance of fine and coarse PM in the induction of deaths. The correlation between fine and coarse PM (r = 0.51) in this study enable separation of the two effects but it was not possible to disentangle their potential effects in a two-pollutant model given their correlation levels. It is unfortunate that, thus far, few studies have reported the results from two-pollutant analyses.

We did not adjust PM_2.5_. Therefore, the adverse effects of PM_2.5–10_ are not likely to be a result of an independent PM_2.5–10_ exposure and are likely to confound with PM_2.5_ levels. While it does not appear as though associations with PM_2.5–10_ are simply due to confounding by PM_2.5_, it is possible that both PM_2.5_ and PM_2.5–10_ are acting as surrogates of a broader mixture of pollution. Englert suggested that the relative sizes of effects attributed to fractions of PM_10_ depend on their relative mass percentages [46]. Although PM_2.5–10_ represented only about 41% of the PM_10_ mass concentration in our study, we found statistically significant association between PM_2.5–10_ and the risk of mortality, which supports a specific effect of this PM fraction. Because some overlap exists in the size ranges between combustion-generated fine particles and mechanically generated particles that generally fall into the coarse range, there is some possibility that the effects detected could be related to fine particles in this intermodal range [27]. 

Epidemiologic studies on the effects of PM_2.5–10_ on daily mortality are limited and inconsistent. Studies conducted in 15 counties in California, Malig and Ostro observed an increased excess risk (ER) of both all-cause mortality (ER per 10 μg/m^3^ = 0.7%, 95% CI = 0.1%–1.5%) and cardiovascular mortality (ER per 10 μg/m^3^ = 1.3%, 95% CI = 0.1%–2.5%) from a 2-day lag in PM_2.5–10_ [27]. Zanobetti and Schwartz found a 0.46% (95% CI = 0.21%–0.71%) increase in total mortality, a 0.32% (95% CI = 0.00%–0.64%) increase in cardiovascular disease (CVD), a 0.84% (95% CI = 0.07%–1.62%) increase in stroke, and a 2.16% (95% CI = 1.14%–3.20%) increase in respiratory deaths for a 10 ug/m^3^ increase in 2-day averaged PM_2.5–10_ levels in 47 US cities [16]. A study conducted in Stockholm, Meister *et al.* found positive association between PM_2.5–10_ and daily mortality, with an ER of 1.68% (95% CI = 0.2%–3.15%) per 10 ug/m^3^ increase in PM_2.5–10_ level7s [31]. Contrary to the US studies, two European studies failed to show significant association between PM_2.5–10_ levels and all cause or cause specific mortality [29,30]. A study conducted in Shanghai, China, Kan *et al.* did not find significant associations of PM_2.5–10_ with mortality outcomes [28]. A study conducted in three Chinese cities within the China Air Pollution and Health Effects Study (CAPES), Chen *et al.* noted a 0.25% (95% CI = 0.08%–0.42%) increase in total mortality, a 0.25% (95% CI = 0.10%–0.40%) increase in cardiovascular disease (CVD), and a 0.48% (95% CI = 0.20%–0.76%) increase in respiratory mortality for a 10 ug/m^3^ increase in 1-day lagged PM_2.5–10_ in the single-pollutant models [21].

Most studies that have reported significant effects on mortality associated with PM_2.5–10_ were conducted in arid areas, including such places as Phoenix, Arizona [47], Coachella Valley, California [48], and Mexico City [49]. In addition, recent studies from southern Europe have explored the effects of windblown Saharan dust, including studies conducted in Rome (Italy), Madrid (Spain), and Barcelona (Spain), which found evidence of an effect of PM_2.5–10_ on daily mortality during Saharan dust days, despite rather moderate particle concentration [50,51,52]. In arid areas, particle dust often originates from the surrounding land, not from local point sources, and particle levels are therefore expected to be more spatially homogeneous [31].

An 11% and 4% increase of all-cause mortality per 10 μg/m^3^ increment in the 3-day moving average (lag 2) concentrations of PM_2.5–10_ were found during the warm and cool days, respectively. The magnitude of PM_2.5–10_ effect estimates reported in our study were generally larger than those noted previously. Although the study methods might have influenced the analysis data, disparities between the findings of our research and that concerning Western populations provide evidence of differential toxicity of PM_2.5–10_ with different components across locations and on various health outcomes. The basis for differences in these studies is not known. In addition, our previous study showed that PM_2.5_ had a statistically significant association with total mortality both on warm and cool days, with an increment of 10 μg/m^3^ with a 4.06% and 3.48% rise in number of total deaths, respectively [53]. However, no significant effects were found between PM_10_ and all-cause mortality. Our study suggested a greater effect of coarse PM on all-cause mortality compared with PM_2.5_ and PM_10_ [54].

Only in recent years have researchers begun to separately address the adverse health effects attributed to PM_2.5–10_, because (1) PM_2.5–10_ were initially considered as potentially less toxic than PM_2.5_ due to their large size and small surface area to mass ratio and (2) it is only recently that PM_2.5_ were measured separately [55]. However, PM_2.5–10_ may plausibly impact health given their deposition in the lungs, high biological content such as organic matter and microbes, and, in urban areas, high content of heavy metals such as Fe and Al [56]. Further, particle agglomerates that are large enough to be in the coarse fraction may contain many ultrafine particles and other attached constituents [52].

Results from toxicological studies showed that on an equal mass basis, coarse and fine PM both produce inflammatory effects, including some evidence that coarse PM may be more inflammatory than fine PM [57,58,59,60]. Controlled human exposure studies have also provided evidence of acute alterations in markers of inflammation, coagulation, and autonomic tone [61,62,63,64,65]. In addition, controlled exposure to concentrated ambient PM_2.5–10_ can also produce increase in neutrophils in healthy humans [62,63]. Such inflammatory responses are a major component of asthmatic disease and may incite or exacerbate other respiratory conditions [66,67].

Major PM_2.5–10_ components vary by region and by season, but typically include Ca, Mg, Al, silicon, and Fe, and primary organic materials such as pollen, spores, and plant and animal debris [18,22,23]. Despite considerable research, the relative toxicity of different constituents of PM_2.5–10_ remain unclear but likely vary dependent upon the components [60]. The origin of chemical pollutants in an urban atmosphere is known to be predominantly attributed to road traffic [12]. Concentrations in urban environments generally are more influenced by transportation than in rural conditions, in which agriculture, other sources such as unpaved and construction sites, and wind are key influences [18]. In our study, the effect of PM_2.5–10_ on daily mortality did not vary by season (no effect modification). The reason for this is unknown. The seasonal pattern of air pollution health effects needs to be further investigated.

The case-crossover study design was proposed by Maclure [33] to study the influence of transient, intermittent exposures on the subsequent risk of rare acute-onset events in close temporal proximity to exposure. This design offers the ability to control many confounders by design rather than by statistical modelling. This design is an adaptation of the case-control study in which each case serves as his or her own referent. Therefore, time-invariant subject-specific variables such as gender, age, underlying chronic disease, or other individual-level characteristics do not act as confounders. In addition, time-stratified approach [36] was found to be effective in controlling for seasonality, time trends, and chronic and slowly varying potential confounders [40,41,42]. In general, the case-crossover design and the general additive model (GAM) approach, which has been the analytic method of choice for studying short-term adverse effects of air pollution since 1990 [68], produced almost identical results [37,38,39].

For a factor to confound the relationship between PM_2.5–10_ levels and daily mortality, it needs to be correlated with both variables. It is unlikely that smoking and other indoor pollutants confound the present association since day to day variations in indoor emissions, including smoking, may not be correlated with coarse PM air pollutants.

Exposure measurement error is a common concern in environmental epidemiology. PM_2.5–10_ levels are typically more spatially heterogeneous than PM_2.5_ due to its higher deposition velocities in the atmosphere for these higher mass particles [26,56]. Concentrations of PM have also been shown to vary across space based on proximity to different sources, making exposure assignment especially difficult given the limited numbers of monitoring stations with data to estimate PM_2.5–10_ [56]. The potential for exposure measurement error in epidemiologic studies based on monitoring stations is likely to be greater for investigating associations of health indicators with PM_2.5–10_ than PM_2.5_ [26]. Larger measurement error relative to PM_2.5_ may be a plausible explanation for weakened associations for PM_2.5–10_ in this study. Measurements of PM_2.5–10_ levels are indirect, estimated through subtraction of PM_2.5_ from PM_10_ concentrations measured at the same monitoring station in this study. While past investigations deemed this a reliable approach to estimating PM_2.5–10_ in urban areas [56], there are inherently errors due to the uncertainty of both filters. PM_2.5–10_ were assigned from fixed, outdoor monitoring stations to individuals to estimate exposure (assuming that exposure was homogeneous encompassing all the studied area). Exposure measurement errors resulting from differences between the population-average exposure and ambient PM_2.5–10_ levels are not avoidable. However, the potential for misclassification of exposure due to the lack of personal measurements of PM_2.5–10_ exposure in this study is of the Berkson-type, known to produce a bias toward the null and an underestimate of the relationship [43,69].

Our study population is homogenous in terms of race compared with populations in other cities. This study was conducted in a tropical city. These facts may restrict somewhat the generalizability of these findings to other locations with different meteorological and racial characteristics. Further, behavior such as air conditioning usage or time spent outdoors may affect personal exposures. This might affect the magnitude of the observed associations compared with other geographical locations.

## 4. Conclusions 

In summary, we found statistically significant association between PM_2.5–10_ and the risk of death for all causes. This finding is not likely to be a result of an independent PM_2.5–10_ exposure as PM_2.5_ is not controlled for in this study. The ecological design of the study precludes the inference of cause and effect. However, these findings reinforce the possible role of PM_2.5–10_ in induction of adverse health effects.

## Figures and Tables

**Table 1:** Mortality counts, weather and air pollutant concentrations in Taipei, Taiwan, 2006–2008.

Parameter	Min	25%	50%	75%	Max	Mean
PM_10_ (μg/m^3^) ^a^	15.33	36.26	48.01	62.28	205.35	52.07
PM_2.5_ (μg/m^3^) ^a^	8.25	19.79	27.75	37.02	117.72	30.65
PM_2.5–10_(μg/m^3^) ^a^	2.79	14.65	19.61	24.78	144.15	21.45
SO_2_ (ppb) ^a^	1.12	3.07	4.05	5.35	11.14	4.32
NO_2_ (ppb) ^a^	3.73	20.66	24.53	29.55	55.51	25.37
CO (ppm) ^a^	0.15	0.53	0.66	0.82	1.73	0.70
O_3_ (ppb) ^b^	6.98	30.19	37.76	49.64	115.43	40.63
Temperature (°C)	9.35	19.67	24.32	28.27	32.78	23.74
Humidity (%)	47.92	66.95	73.21	79.28	94.19	73.02
Total deaths per day	2	11	14	17	32	14.29
Respiratory deaths	0	1	1	2	7	1.51
Circulatory death	0	2	4	5	11	3.93

Min: minimum value; Max: maximum value; ^a^ 24-h average; ^b^ daily maximal 8-h average.

**Table 2:** Correlation coefficients among air pollutants.

Variable	PM_2.5_	PM_2.5–10_	SO_2_	NO_2_	CO	O_3_
PM_10_	0.91 *	0.83 *	0.63 *	0.47 *	0.45 *	0.44 *
PM_2.5_	1.00	0.51 *	0.61 *	0.52 *	0.51 *	0.44 *
PM_2.5__–__10_	-	1.00 *	0.47 *	0.27 *	0.24 *	0.32 *
SO_2_	-	-	1.00	0.49 *	0.45 *	0.31 *
NO_2_	-	-	-	1.00	0.88 *	0.25 *
CO	-	-	-	-	1.00	0.14 *
O_3_	-	-	-	-	-	1.00

^*^ *p* < 0.05.

**Table 3:** Adjusted odds ratios (AORs) and 95% confidence intervals (CIs) for daily mortality for each interquartile range increase ^a^ of PM _2.5–10_ in Taipei, Taiwan, 2006–2008, stratified by temperature.

Temperature	Total Deaths AOR (95% CI)	Respiratory Disease AOR (95% CI)	Circulatory Disease AOR (95% CI)
≥23 °C (617 days)			
Without adjustment ^b^	1.11 (1.06–1.17)	1.11 (0.95–1.29)	1.10 (1.01–1.21)
Adjusted for SO_2_	1.11 (1.06–1.17)	1.10 (0.97–1.29)	1.10 (1.00–1.20)
Adjusted for NO_2_	1.08 (1.03–1.14)	1.07 (0.91–1.27)	1.07 (0.97–1.18)
Adjusted for CO	1.07 (1.02–1.12)	1.06 (0.90–1.25)	1.06 (0.97–1.17)
Adjusted for O_3_	1.12 (1.07–1.17)	1.10 (0.94–1.29)	1.11 (1.01–1.22)
<23 °C (479 days)			
Without adjustment ^b^	1.04 (1.01–1.07)	1.03 (0.93–1.13)	1.05 (1.00–1.11)
Adjusted for SO_2_	1.05 (1.02–1.09)	1.04 (0.94–1.16)	1.07 (1.01–1.14)
Adjusted for NO_2_	1.01 (0.98–1.04)	1.00 (0.91–1.11)	1.03 (0.97–1.09)
Adjusted for CO	1.02 (0.99–1.05)	1.02 (0.92–1.13)	1.03 (0.97–1.10)
Adjusted for O_3_	1.04 (1.01–1.07)	1.03 (0.93–1.13)	1.06 (1.00–1.11)

^a^ Calculated for an interquartile range increases of PM_2.5–10_ (10.13 μg/m^3^) and adjusted for temperature and humidity; ^b^ Single pollutant model.
